# Anticancer Activity, Mechanism, and Delivery of Allyl Isothiocyanate

**DOI:** 10.3390/bioengineering9090470

**Published:** 2022-09-14

**Authors:** Ammar Tarar, Sarah Peng, Soha Cheema, Ching-An Peng

**Affiliations:** 1 Department of Chemical and Biological Engineering, University of Idaho, Moscow, ID 83844, USA; 2 Department of Chemistry, University of Washington, Seattle, WA 98195, USA; 3 Department of Pharmacy, University of Lahore, Lahore 54590, Pakistan

**Keywords:** allyl isothiocyanate, sinigrin, myrosinase, glucosinolate, anticancer, mechanism of action, drug delivery

## Abstract

Allyl isothiocyanate (AITC) is a phytochemical that is abundantly present in cruciferous vegetables of the *Brassicaceae* family, such as cabbage, broccoli, mustard, wasabi, and cauliflower. The pungent taste of these vegetables is mainly due to the content of AITC present in these vegetables. AITC is stored stably in the plant as its precursor sinigrin (a type of glucosinolate), which is physically separated from myrosin cells containing myrosinase. Upon tissue disruption, myrosinase gets released and hydrolyzes the sinigrin to produce AITC and by-products. AITC is an organosulfur compound, both an irritant and toxic, but it carries pharmacological properties, including anticancer, antibacterial, antifungal, and anti-inflammatory activities. Despite the promising anticancer effectiveness of AITC, its clinical application still possesses challenges due to several factors, i.e., low aqueous solubility, instability, and low bioavailability. In this review, the anticancer activity of AITC against several cancer models is summarized from the literature. Although the mechanism of action is still not fully understood, several pathways have been identified; these are discussed in this review. Not much attention has been given to the delivery of AITC, which hinders its clinical application. However, the few studies that have demonstrated the use of nanotechnology to facilitate the delivery of AITC are addressed.

## 1. Introduction

**Figure 1:**
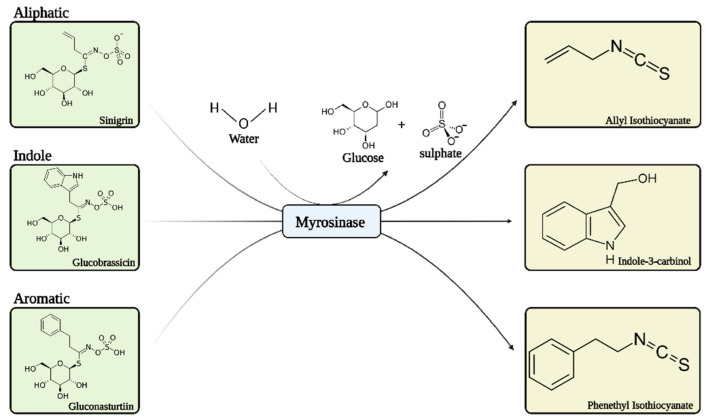
Chemical structure of aliphatic, indole, and aromatic GLs and their corresponding products after catalytic hydrolysis by myrosinase.

Glucosinolates (GLs) are secondary metabolites abundantly present in the cruciferous vegetables of the *Brassicaceae* family, i.e., horseradish, broccoli, cabbage, watercress, brussels sprouts, mustard, cauliflower, etc. [1]. There are several types of GLs, but they share a common structure of a β-D-thio-glucosylated moiety attached to variable side chain R (derived from α-amino acid) and the sulfonated aldoxime moiety [2]. Hundreds of GLs have been identified and can be classified into three categories based on the structure of their precursor amino acids: aliphatic, aromatic, and indole GLs (Figure 1) [3]. The level and composition of GLs in plants are influenced by several factors such as climate, genotype, and cultivation conditions; even different parts of plants determine the level of GLs. For instance, indole GLs are more abundant in growing leaves and young shoots [4]. During the past two decades, GLs have been reported to have several pharmacological properties, such as antibacterial, anticancer, antifungal, anti-inflammatory, and antioxidant activities [5]. Although GLs are biologically inactive, they can be hydrolyzed by β-thioglucoside hydrolases (myrosinase) to produce bioactive compounds (e.g., thiocyanates, isothiocyanates, nitriles, etc.) that are responsible for these pharmacological activities. Each GL is hydrolyzed to a different isothiocyanate, e.g., glucoraphanin is hydrolyzed by myrosinase to produce sulforaphane (SFN), which was first isolated from broccoli. Glucobrassicin and gluconasturtiin are hydrolyzed to produce indole-3-carbinol and phenethyl isothiocyanate, respectively, as shown in Figure 1. Sinigrin, a common GL, is hydrolyzed in a similar manner to produce allyl isothiocyanate (AITC), which is abundant in mustard oil.

**Figure 2:**
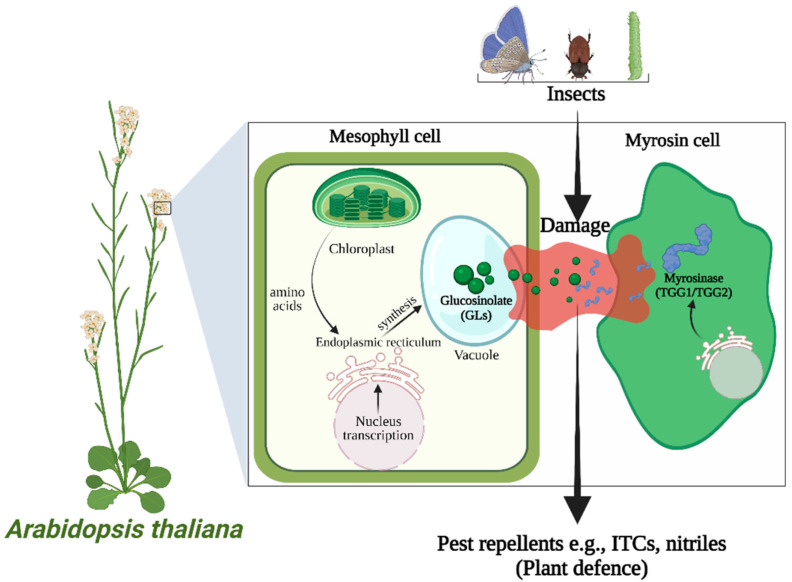
Schematic illustration of the plants’ defense mechanism. The attack of insects leads to the collapse of glucosinolates-containing cells (mesophyll cells) and myrosin cells, which allows myrosinase to come into contact with GLs and hydrolyzes to produce toxic products, i.e., ITCs and nitriles.

AITC is stored stably as sinigrin (allyl glucosinolate or 2-propenyl glucosinolate), which is a type of aliphatic GL. It is abundantly present in brussels sprouts, broccoli, and mustard seed (seeds of *Brassica nigra*), giving pungency to these plants [3]. Myrosinase cleaves the glucose moiety of sinigrin to produce intermediate aglycone, which is unstable and rearranges itself spontaneously to AITC [6]. The sinigrin and myrosinase system is part of the plants’ defense mechanism and is present in different compartments of a plant cell; they are physically separated from each other to prevent self-intoxication. When plant tissue is masticated, ruptured, eaten, or damaged in other ways, myrosinase interacts with sinigrin to produce hydrolysis products that are irritants and toxic [7], hence serving as a pest repellent (as illustrated in Figure 2).

Epidemiological studies suggest that the consumption of cruciferous vegetables could decrease the risk of colorectal cancer. Although cruciferous vegetables eaten by humans contain glucosinolate and myrosinase, which can be readily hydrolyzed to produce ITCs, cooking these vegetables will inactivate the enzymatic activity of myrosinase. Human gut microflora also has myrosinase-like enzymes that have the ability to hydrolyze the glucosinolates; however, little is known about this type of enzymatic activity to produce pharmacological effects.

Many studies have been published regarding the pharmacological properties of isothiocyanates (ITCs), including their ability to inhibit the growth of cancer. Among them, one of the promising anticancer agents is AITC, which exhibits anticancer activity through several mechanisms, such as cell cycle arrest, inducing apoptosis, and decreasing metastasis and invasion [8]. In this regard, several studies have been published, and in this review, the anticancer activity of AITC against various carcinomas is discussed and divided into subsections: principal findings, the anticancer mechanism, and the delivery of AITC.

## 2. Anticancer Activity of AITC

**Figure 3:**
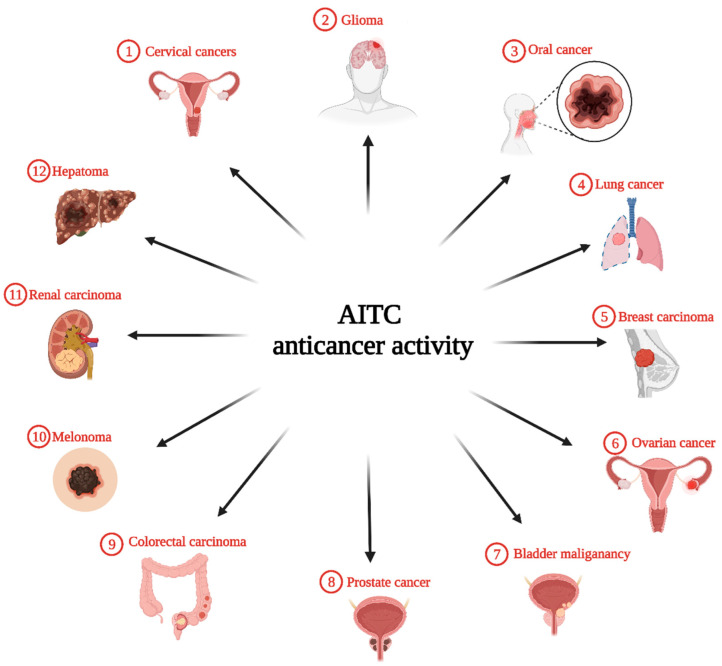
Schematic drawing of anticancer activity of AITC against various cancer types reported in the literature.

AITC has been reported as an anticancer agent for several carcinomas, from cervical cancer to hepatoma, as shown in Figure 3. In this section, we summarize and discuss the anticancer activity of AITC against several cancer cell types reported in the literature. A summary is also displayed in Table 1.

### 2.1. Cervical Cancer

Qin et al. revealed that AITC inhibited cell viability and induced apoptosis in the human cervical cancer HeLa cell line in vitro when treated with 45 μM for 72 h. Furthermore, they found that the apoptosis rate increased in a dose-dependent manner. B-cell lymphoma-2 (Bcl-2) expression decreased, and Bcl-2-associated X protein (Bax) expression increased, leading to a continuous decrease in the ratio of Bcl-2/Bax proteins, which is a key indicator of apoptosis. Thus, AITC may have instigated this imbalance between the Bcl-2/Bax expression ratio to inhibit the cell viability of HeLa cells and induce apoptosis [34].

### 2.2. Malignant Glioma

The anticarcinogenic properties of AITC in malignant glioma GBM 8401 cells of the human brain were reported to be due to mitochondria-dependent pathway apoptosis. The IC_50_ value of 9.25 ± 0.69 μM was determined after 24-hour treatment of AITC. This decrease in cell viability was mediated by G2/M phase cell cycle arrest due to a decrease in CDK1, cyclin A, and cyclin B activity [18].

### 2.3. Cisplatin-Resistant Oral Cancer Cells

AITC decreased the cell viability of cisplatin-resistant oral cancer (CAR) cells significantly in a dose- and time-dependent manner, with an IC_50_ value of ~30 μM after 48 h. The inhibitory effect was due to DNA fragmentation and the downregulation of p-AKT and p-mTOR. This promoted the mitochondria-dependent apoptotic pathway by augmenting caspase-3 and caspase-9 activity [41].

### 2.4. Non-Small Lung Carcinomas

The anticancer mechanisms of AITC in human A549 and H1299 non-small cell lung cancer (NSCLC) cells were reported to be due to S and G2/M cell cycle arrest, γH2AX, FANCD2 foci, and ATM/ATR-mediated checkpoint responses that induced replication stress in NSCLS cells. AITC showed more effective inhibition, with IC_50_ values of 5 and 10 μM for H1299 and A549 cells, respectively, compared to phenyl isothiocyanate (PITC), which showed 7.5 and 15 μM for H1299 and A549 cells. Moreover, the study also reported that the tumor cells became more sensitized to radiation therapy after treatment with ITCs, and combination therapy resulted in CI (combination index) values of less than 0.7 [32]. In a similar study that showed enhanced anticancer activity, AITC was combined with sulforaphane (SFN) to inhibit A549 cells [33].

### 2.5. Breast Cancer

AITC has been reported for its chemopreventive mechanism and induction of apoptotic cell death in triple-negative MDA-MB-468 human breast cancer cells in vitro through ERK-modulated intrinsic signaling and G2/M phase arrest analyzed through flow cytometry. Intrinsic apoptosis-associated factors, i.e., production of reactive oxygen species (ROS) and loss of mitochondrial membrane potential (∆Ψm), appeared in the AITC-treated MDA-MB-468 cells [26]. Similar results were obtained from another study in which AITC inhibited the growth of MCF-7 (estrogen receptor positive) and MDA-MB-23 (triple-negative) human breast cancer cells in a dose-dependent manner, with an IC_50_ value of ~5 μM [27]. However, in another study, AITC did not show any antiproliferative activity against MDA-MB-23 human breast cancer cells. Sayeed et al. reported that 10 μM of AITC could not inhibit proliferation but, instead, increased the growth slightly [42]. Nevertheless, both studies showed that AITC was cytotoxic in a dose-dependent manner on the MCF-10A human breast epithelial cell line [42]. We believe that the discrepancy in results of MDA-MB-23 is due to the significant difference in the inoculation of cells in both groups. For instance, Bo et al. inoculated 2 × 10^5^ cells/well in a 12-well plate (seeding density ~5000 cells/cm^2^) and Sayeed et al. inoculated 2 × 10^4^ cells/well in a 96-well plate (seeding density ~62,000 cells/cm^2^). Because of the high seeding density in the latter study, 10 μM of AITC could not inhibit malignant cell growth.

### 2.6. Ovarian Cancer

Barnett et al. reported the tumor growth inhibitory properties of dietary isothiocyanates (AITC and PITC) on ovarian cancer by inducing the replication of the stress-mediated DNA damage response. They found that AITC promoted a more cytotoxic effect and inhibited ovarian cancer cell growth by showing antimetastatic effects in both platinum-resistant and platinum-sensitive cancer cell lines [43].

### 2.7. Bladder Cancer Cells

Bhattacharya et al. reported the inhibition of bladder cancer growth treated with AITC (IC_50_ of 2.7–3.3 μM) in contrast to normal human bladder epithelial cells, to which AITC is less toxic and shows IC_50_ of 69.4 μM [21]. In their further studies, mustard seed powder (MSP-1) was used to inhibit bladder cancer growth, development, and muscle invasion. MSP-1 stores AITC in its inactive form, sinigrin, which is hydrolyzed to AITC in the presence of endogenous myrosinase enzymes. During their in vitro experiments, hydrated MSP-1 caused G2/M phase cell cycle arrest and apoptosis in rat bladder cancer AY-27 cells and human bladder cancer UM-UC-3 cells [19]. The same research group further demonstrated that AITC induced cell death through mitochondria-mediated apoptosis, which was evident from the release of cytochrome c into the cytoplasm from mitochondria, the activation of caspase 3 and caspase 9, and mitotic arrest via Bcl-2 phosphorylation at Ser-70, caused by JNK (c-Jun N-terminal kinase) [20]. TP53 is the most common mutation in bladder cancer, and it is commonly detected in situ carcinomas. The AITC effect on bladder cancer cells depends on the expression of the TP53 gene, which has been investigated using the RT4 cell line (wild-type TP53) and the T24 cell line (mutated TP53 gene). It was found that AITC was able to induce primary DNA damage in both wild-type and mutant cell lines. However, increased apoptosis and necrosis were observed in wild-type TP53 expressing RT4 cancer cells [23].

### 2.8. Prostate Cancer

Xiao et al. demonstrated that AITC caused a cytotoxic effect by causing G2/M phase cell cycle arrest and inhibited proliferation of both androgen-independent (PC-3) and androgen-dependent (LNCaP) human prostate cancer cells by using an IC_50_ of ~17 μM. They also reported that AITC failed to cause G2/M phase arrest or the induction of apoptosis in normal prostate epithelial cell line (PrEC) cells. There was a marked decrease in the level of proteins such as Cdk1 (32–50%), Cdc25B (44–48%), and Cdc25C (>90%) after 24 h of treatment with a 20 μM concentration of AITC in PC-3 and LNCaP cells, but the reduction of cyclin B1 protein levels (~45%) was seen only in LNCaP cells. Antiapoptotic protein levels (Bcl-2) were greatly reduced after 24 h of treatment with 20 μM of AITC in both PC-3 and LNCaP cell lines, while only LNCaP cells showed a ~58% reduction in Bcl-XL protein levels [11]. In the most recent study, AITC was reported to induce protective autophagy through BECN1 (beclin-1) upregulation in PC3 human prostate cancer cells [13].

### 2.9. Colorectal Cancer

AITC has been reported as an anticancer agent against human colorectal cancer in several studies. Lai et al. reported the suppression and migration of EGF (epidermal growth factor)-stimulated HT29 human colorectal cell line cells using a transwell cell invasion assay. They showed that 5 and 10 μM of AITC inhibited cell migration by 48% and 81%, respectively, and significantly decreased the invasion of HT29 cells resulting from the downregulation of MMP-2/-9 (matrix metalloproteinase-2/9) and MAPKs (mitogen-activated protein kinases) [36]. To further understand the mechanism, the group conducted experiments and revealed that AITC encourages mitochondria-related signaling, including Endo G, Apaf-1, cytochrome c, and AIF signaling. They concluded their study by stating that AITC induced apoptosis through a mitochondrial-dependent pathway and endoplasmic reticulum stress [44]. Similar findings were reported by Chiang et al.; they showed the time-dependent reduction in cell viability of HT29 cells after treatment with 5, 10, and 20 μM of AITC [45]. In another study, Musk and Johnson demonstrated that AITC displayed both cytostatic and cytotoxic properties by damaging cellular and plasma membrane systems and were toxic to HT29 cells in vitro [37]. AITC also showed anticancer activity against human metastatic colorectal adenocarcinoma cells. Lau et al. reported that the viability of Caco-2, SW620, and COLO 201 cells decreased to 50%, 42%, and 65%, respectively, after treatment with 50 μM of AITC for 72 h [38].

### 2.10. Metastatic Melanoma Cells

The effect of AITC on human melanoma A375 cells and murine melanoma B16-F10 cells was also investigated. Cells showed reduced viability with IC_50_ values of 12.0 ± 0.7 and 14.9 ± 3.7 μM for A375 and B16-F10 cells, respectively, in 48 h of treatment. AITC induced apoptosis by regulating the expression of various apoptotic cascades (extrinsic-FAS, FASLG; intrinsic-BAK1, CASP9, and p53-dependent) and decreased the activity of various HATs (acetyltransferases), HDACs (histone deacetylases), and HMTs (histone methyl transferases), thus effecting lysine acetylation and methylation [39].

### 2.11. Renal Cell Carcinomas

AITC was found to inhibit renal carcinoma cell GRC-1 proliferation and induce apoptosis in a dose- and time-dependent manner. AITC induced increased Bax expression while decreasing the Bcl-2 expressions at both mRNA and protein levels. Thus, an inverse relation between AITC and the Bcl-2/Bax ratio was found, i.e., increasing the concentration of AITC decreased the Bcl-2/Bax ratio, resulting in an anticarcinogenic effect on the GRC-1 cells [40]. Bax and Bcl-2 are apoptotic-associated proteins and are antagonist pairs that can modulate apoptosis; the increased Bcl-2/Bax ratio was found to suppress apoptosis and vice versa [46].

### 2.12. Leukemia

AITC inhibited the growth of human leukemia cells (HL60) and human myeloblastic leukemia-1 (ML-1) cells in vitro with a median growth inhibitory concentration (GC50) of 2.56 ± 0.11 μM. AITC and its cysteine conjugate were responsible for growth arrest and the inhibition of DNA synthesis. Further studies on leukemia were carried out by Zhang et al., in which they explored the AITC effect on human promyelocytic acute leukemia HL60/S cells and its doxorubicin-resistant derivative HL60/AR cells. The study was comprehensive in evaluating different ITCs’ effects on different cancer cell models. The IC_50_ value of 2.0 ± 0.3 and 4.1 ± 0.4 for HL60/S and HL60/AR cells, respectively, were reported after 3 h of AITC treatment [47].

## 3. AITC Anticancer Mechanisms

**Figure 4:**
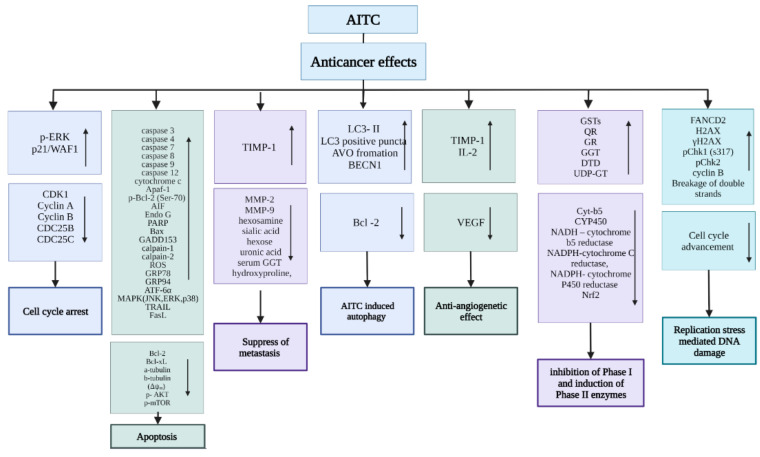
Summary of the mechanisms of the anticancer effects of AITC. The arrow facing upward shows the upregulation of genes, and the arrow facing downward shows the downregulation of specific genes.

Although the full mechanism of AITC-induced antineoplastic effects is still not understood, several pathways have been identified and are given below. A summary of the anticancer mechanisms of action is given in Figure 4.

### 3.1. Stimulation of Cell Cycle Arrest

The cell cycle consists of four major phases: the G1 phase (growth phase 1), the S phase (DNA replication), the G2 phase (cell grows rapidly and prepares itself for mitosis), and the M phase (cell division) [48]. Cyclins regulate the progression of the cell cycle from one phase to another by building complexes with cyclin-dependent kinases (CDKs) and are inhibited by CDK inhibitors (such as p21 and p27). In cancer, the deregulation of cyclin or CDK expression levels can lead to uncontrolled cancerous cell division [49]. Cell cycle arrest is a temporary or permanent regulatory process that halts the cell cycle progression so that cells no longer divide uncontrollably [50]. Hasegawa et al. reported the induction of cell cycle arrest at the G2 and M phases in HeLa cells after 16-hour treatment with 10 μM of AITC, but the mechanism was not so clear back then [51]. Chen et al. reported a decrease in CDK1 activity, cell viability, and cyclin A and cyclin B levels after 24-hour treatment with 10 μM of AITC in human malignant glioma GBM 8401 cells, which ultimately resulted in G2/M phase cell cycle arrest [18]. The marked decrease in the level of proteins, i.e., Cdc25B (44–48%), Cdc25C (>90%), and Cdk1 (32–50%), after 24 h of treatment with a 20 μM concentration of AITC was observed in both androgen-independent (PC-3) and androgen-dependent (LNCaP) human prostate cancer cells [11]. Later, it was also confirmed during another study of AITC-treated PC-3 cells that showed the downregulation of Cdc25B, cyclin B1, and Cdc25C expressions by 45%, 44%, and 90%, respectively, resulting in a significant reduction of G2/M phase progression. This suggests that reduced mitotic activity may be due to inactive CDK1 and cyclin B complexes [12]. Moreover, AITC induced G2/M phase cell cycle arrest in MCF-7, MDA-MB-231, and MDA-MB-468 human breast adenocarcinoma cells through the upregulation of p-ERK and p21/WAF1, which is a cyclin-dependent kinase inhibitor (CKI), and the downregulation of CDK1 and cyclin B proteins [26,52].

Savio et al. researched possible AITC effects on TP53 gene expression in two transitional bladder cancer cell lines, RT4 (with wild-type TP53) and T24 (mutated TP53 gene). Cell cycle analysis indicated a reduced number of RT4 cells in the S phase in response to treatment with AITC (at 0.005, 0.0625, 0.0725, and 0.0825 μM), and a slight increase in G1 phase cells was also observed. However, T24 cells were reduced in numbers in the G1 phase (at 0.0725 and 0.0825 μM concentration of AITC) and the S phase (at 0.0625 and 0.0825 μM concentration of AITC), followed by an increase in the number of cells in the G2 phase, signifying G2/M cell cycle arrest [23]. Bhattacharya et al. studied low-dose treatments of AITC in human bladder carcinoma (IC_50_ = 2.7 μM) and rat bladder carcinoma (IC_50_ = 3.3 μM), resulting in G2/M phase arrest and inhibiting the proliferation of bladder carcinogenic cell lines through the downregulation of cyclin B1 activity. This low-dose treatment with AITC turned out to be the strongest one against bladder carcinomas, but the reason for this is not known. However, AITC showed much lower toxicity in the case of normal human urothelial cells (HUCs) (IC_50_ = 69.4 μM) [21].

The combined dose-dependent treatment of 12.5 μM AITC and 10 μM SFN (sulforaphane) significantly enhanced G2/M phase cell cycle arrest by 47%, decreased the G0/G1 population by 37%, lowered the S phase population, and led to an increase in p21 protein expression in non-small cell lung carcinoma cells (A549 cells) [33]. After 4-h treatment with 20 μM, AITC induced G2/M phase cell cycle arrest by decreasing vital Cdc25B and Cdc25c protein phosphatase expression levels (~ 0.5 folds) in human colorectal adenocarcinoma SW620 cells in vitro and inhibited further tumor cell division [38]. Similarly, a study conducted by Smith et al. demonstrated that 25% of HT29 colorectal cells were arrested in the M phase of the cell cycle after treatment with AITC [35]. Moreover, AITC caused G0/G1 phase cell cycle arrest in both MDA-MB- 231 and MCF-7 cell lines [27] and exhibited S phase and G2/M phase cell cycle arrest in non-small cell lung cancer (NSCLC); additionally, tumor cells became more sensitized to radiation therapy [32].

### 3.2. Induction of Apoptosis

Programmed cell death occurs in a multicellular organism and is known as apoptosis. It is characterized by shrinkage of the damaged cell when chromatin starts to condense and cellular blebbing and organelle disintegration begin, which is then followed by DNA fragmentation and the formation of apoptotic bodies. Apoptosis helps in getting rid of damaged and injured cells that cannot be further repaired [53]. Apoptosis can be regulated through the adjustment of various pathways, such as the intrinsic or mitochondrial pathway, the extrinsic pathway, and the caspase-independent pathway [54,55]. Many studies have shown apoptosis in response to AITC treatment by targeting different checkpoints in these pathways. These include the upregulation of Bax (proapoptotic protein) and the downregulation of Bcl-2 (antiapoptotic protein), the activation of MAPKs such as the c-Jun N-terminal kinase (JNK), the extracellular signal-regulated kinase (ERK), and p38 [54]. Moreover, there will be the activation of caspase 3, caspase 9 activities, and enhanced levels of cytochrome c, AIF, Endo -G, and Apaf-1 [5]. The 24-hour treatment with 20 μM of AITC in PC-3 and LNCaP cells significantly increased caspase 3 activity in PC-3 for the first 1 to 4 h only. However, LNCaP cells’ caspase activity remained elevated for the complete period of the experiment. Antiapoptotic protein levels (Bcl-2) were greatly reduced in both PC-3 (31%) and LNCaP (68%) cell lines, while only LNCaP cells showed a ~58% reduction in Bcl-XL protein levels. Meanwhile, Bax and BID (proapoptotic proteins) levels remained unaffected [11]. Similarly, up to 70% reduced Bcl-2 expression and the cleavage of p23.BID to the p15 fragment was seen in AITC-treated mice models, resulting in increased apoptosis of tumor cells, although Bcl-XL and Bax expressions remained unaffected [12].

In another study, bladder cancer growth was inhibited to 34.5% and muscle invasion was inhibited to 100% in an orthotopic rat model. AITC induced apoptosis by activating caspase 3 activity, cleaving PARP (poly adenosine diphosphate ribose polymerase), and downregulating VEGF (vascular endothelial growth factor) after oral ingestion of MSP-1 (mustard seed powder-1) at 71.5 mg/kg [19]. In addition to the above-mentioned factors, when AITC is given as an N-acetylcysteine conjugate (NAC-AITC), it causes the downregulation of α- and β-tubulin [22]. Extrinsic and intrinsic pathways play a very important role in the induction of apoptosis. The extrinsic pathway leads to apoptosis through the activation of TRAIL (TNF-related apoptosis-inducing ligand), FasL (Fas/Fas ligand), and caspase 8 activity. The intrinsic pathway, also known as the mitochondrial pathway, promotes cytochrome c, (Apaf-1), caspase 9, and caspase 3 activities [56,57]. AITC decreased Bcl-2 levels and upregulated p-ERK, p-Bcl-2 (Ser-70), cytochrome c, Apaf-1, caspase 3, and caspase 9 activities in MDA-MB-231, MCF-7, and MDA-MB-468 cells [26]. According to a study by Bo et al., AITC enhanced the levels of AIF, Endo G, cytochrome c, PARP, caspase 9, caspase 12, caspase 7, and Bax protein in MCF-7 cells. In contrast, in MDA-MB- 231 cells, AITC enhanced the levels of calpain-2, caspase 3, caspase 12, caspase 7, GADD153, catalase, AIF, Endo G, and PARP. Moreover, it also enhanced the level of reactive oxygen species (ROS) and Ca^2+^ while decreasing the mitochondrial membrane potential (ΔΨm) in both cell lines (MDA-MB- 231 and MCF-7). Some studies have suggested that factors involved in ROS production could lead to ER stress and, thus, cause ER Ca^2+^ to be released. From these explanations, we can infer the involvement of AITC in apoptotic induction in both MDA-MB- 231 and MCF-7 cells [27]. Increased Bax protein and decreased Bcl-2 expression were observed in Ehrlich ascites tumor (EAT) cells after inducing AITC, leading to apoptosis [31]. AITC lowered cell viability and encouraged apoptosis through mitochondria-related signaling in HT29 cells, as evidenced by the increased Endo G, AIF, cytochrome c, Apaf-1, GRP78, GADD153, calpain 1, GRP94, ATF-6α, and caspase-4 levels. AITC exhibited caspase 3 and caspase 9 activities and ROS production; loss of membrane potential (ΔΨm) and cytosolic Ca^2+^ release were also observed in HT29 cells [44]. The 24 and 48 h treatments of AITC decreased human CAL27 cisplatin-resistant oral cancer cell (CAR cell) viability by inhibiting p-AKT and p-mTOR and promoted the mitochondria-dependent apoptotic pathway by augmenting Bax, caspase-3, caspase-9, Apaf-1, and cytochrome c activities [41]. Through either rapid interaction of AITC or PEITC, their cysteine conjugates with HL60 cells and M-1 cells within the first hour of culture or exposure to ITCs and is released from the cysteine conjugate in the initial 3 h of culture and directed toward cell toxicity and growth arrest. Inhibition of DNA synthesis, macromolecule synthesis, protein synthesis, and RNA synthesis were developed as a commitment to apoptosis in the initial 24 h [14]. AITC upregulated caspase 3 and caspase 9 activities and elicited mitochondrial apoptosis by enhancing AIF, cytosolic cytochrome c, and Endo G levels in a time-dependent manner to GBM 8401 cells [18]. AITC induced cell death in human bladder cancer through mitochondria-mediated apoptosis, which was evident from the release of cytochrome c into the cytoplasm from mitochondria, the activation of caspase 3 and caspase 9, and the formation of TUNEL-positive cells. Moreover, it also produced mitotic arrest by binding directly to cysteine residues and causing the increased ubiquitination and degradation of α- and β-tubulin [20]. At higher concentrations of AITC, the upregulation of Bax protein expression and the downregulation of Bcl-2 protein expression were observed; this led to a decrease in the ratio of Bcl-2/Bax proteins, which created an imbalance between the Bcl-2/Bax expression, resulting in decreased cell viability by inducing the apoptosis of HeLa cells [34]. Similar trends of decreased Bcl-2/Bax proteins were observed in the renal carcinoma cell GRC-1 after treatment with AITC, leading to cell death in a dose-dependent manner [40].

### 3.3. Suppression of Metastasis

Metastasis can be defined as the spread of cancer from its primary site of origin to other various regions of the body. Metastasis is responsible for about 90% of deaths in cancer patients [58]. Many drugs can be used for cancer treatment, but scientists are still looking for antimetastatic drugs with high efficacy and minimum toxicity that prove to be beneficial in the suppression of metastasis activity. Metastatic cancer is characterized by increased cellular proliferation, adhesion, invasion, and migration and the degradation and disorganization of the extracellular matrix, which further promotes angiogenesis and inflammation [19,59]. The overexpression of matrix metalloproteinases (MMP-2 and MMP-9) has a direct impact on the degradation of the extracellular matrix, thereby imposing an increase in metastatic terror [60,61,62]. In a study, 5 and 10 μM of AITC appreciably reduced the invasion and migration of HT29 cells induced by EGF, lowered the MMP-2 and MMP-9 protein expressions, and increased the TIMP-1 expression [36]. Similarly, MMP-2/-9 activity was decreased in SK-Hep1 human hepatoma cells after treatment with NAC-AITC and AITC in a dose-dependent manner. Therefore, AITC leads to a decrease in cell proliferation invasion, adhesion, and migration, which confirms its antimetastatic effects [36].

In another study, when 25 mg/rat single dose of DMBA was injected subcutaneously into the mammary tissues of female Sprague–Dawley rats, the levels of glycoprotein components (hexosamine, sialic acid, and hexose) were evaluated. Glycoproteins are formed from the covalent attachment of oligosaccharide chains to amino acids and can be associated with cellular adhesion. Therefore, the level of these sugar moieties in glycoproteins was expected to be linked with the metastatic status of cancer cells [36]. Increased levels of hexosamine, sialic acid, and hexose in plasma, liver, and mammary tissues were observed. Oral administration of AITC in DMBA-injected rats lowered the levels of glycoprotein components back to their original concentrations and implied the antimetastatic effects of AITC by inhibiting abnormal glycosylation [28].

### 3.4. AITC-Induced Autophagy

Autophagy is the body’s natural process of cleaning out damaged, injured, and dysfunctional cells while reproducing new and healthy cells. After the previous induction of protective autophagy through BITC [63] and SFN [64], Chen et al. studied the AITC potential of inducing protective autophagy in prostate cancer cells.

AITC-treated Rv1 and PC3 cells showed the upregulation of BECN1 and an increased BECN1/Bcl -2 expression ratio, which played a crucial role in the regulation of autophagy and was involved in the formation of autophagosomes [65]. LC3-II proteins are a standard marker for the activation of the autophagy pathway [64]. AITC-treated PC3 cells and Rv1 cells showed a 23.33% and 15.78% increase, respectively. Time- and concentration-dependent treatment of AITC showed the formation of acidic vesicular organelles (AVOs) and confirmed the induction of autophagy through the transformation of autophagosomes to auto phagolysosomes when fused with lysosomes. It was also observed that the treatment of Baf A1 (autophagy inhibitor) inhibits the formation of AVOs caused by the treatment of AITC. Furthermore, the pretreatments with 20 μM of Z-VAD-FMK to AITC-treated cells reversed the decrease in cell viability. This confirms that AITC induces protective autophagy in prostate cancer cells [13].

### 3.5. Antiangiogenetic Effect

Angiogenesis is a physiological procedure that is involved with the regeneration of new blood vessels from already developed or pre-existing vasculature. Vascular endothelial growth factor (VEGF) is the principal mediator of this process. It is crucial for wound healing, repair, and growth but also plays an important role in many other pathological conditions, such as tumor growth. Blood is rich in oxygen and other nutrients that are required for tumor growth and development. The blood vessel carries these nutrients to the tumor by receiving chemical signals, helping them grow [65]. In 1971, J. Folkman, for the first time, provided the hypothesis that solid tumors cause the growth of new blood vessels and are angiogenesis dependent. Later on, this hypothesis was proved by many experiments involving genetic methods [66,67]. Since angiogenesis is a central process in tumor progression and metastasis, antiangiogenic or angiogenesis inhibitors are required to stop angiogenesis by cutting its oxygen and nutrient supply [67].

At the concentration of 10 μM, AITC inhibited peritoneal and corneal angiogenesis by decreasing VEGF production to about 40% in the peritoneum cavity of mice transplanted with EAT cells. AITC also inhibits neovascularization in chick eggs, as evidenced by the chorioallantoic membrane (CAM) assay, mouse peritoneum, and rat cornea [31]. Furthermore, AITC-treated B16F-10 cells showed reduced VEGF mRNA levels and the downregulation of TNF-α, IL-6, IL-1β, and GM-CSF and the upregulation of TIMP and IL-2, confirming the anti-angiogenic effect of AITC [68].

### 3.6. Inhibition of Phase I and Induction of Phase II Enzymes

Upon entering the human body, toxic carcinogens are subjected to metabolism by the following two processes, known as phase I metabolism and phase II metabolism. Phase I metabolism occurs via oxidation, reduction, and hydrolysis processes through the involvement of cytochrome P450 enzymes (CYPs) [69]. They usually deactivate, or in certain cases, activate the pro-carcinogens to their active forms, which can bind directly with RNA, DNA, and protein. AITC inhibits neoplastic carcinogenesis by modifying the level of CYPs through a reasonable mechanism [69]. Phase II metabolism plays a very protective role that involves the conjugation of products from phase I metabolism with endogenous ligands such as glutathione S-transferases (GSTs), nicotinamide adenine dinucleotide phosphate, and (UDP)-glucuronosyltransferase to form hydrophilic products that are then excreted or eliminated through urine or bile from the body [69,70]. Many researchers are in favor of utilizing the mechanism of phase II induction for detoxification and phase I inhibition for blocking damaging and deadly carcinogen activation [71,72,73]. When administered orally at the concentration of 40 μmol/kg/day, AITC significantly enhanced GST and QR activities, thereby inhibiting bladder cancer [74].

Increased levels of cytochrome b5 and CYP450 (phase I enzymes) and decreased levels of GR and GST (phase II enzymes) were observed in the liver and mammary glands of DMBA-induced rats. When AITC was given orally to DMBA-induced rats, it regulated the level of these phase I and II enzymes and prevented tumor occurrence and growth, most specifically at a dose of 20 mg/kg of body weight [29]. Increased levels of phase I enzymes such as NADH–cytochrome b5 reductase, NADPH-cytochrome C reductase, and NADPH- cytochrome P450 reductase and decreased phase II enzymes such as GGT, DTD, and UDP-GT were regularized to their optimum levels upon the addition of AITC to DMBA-induced rats [30].

### 3.7. Induction of Replication Stress-Mediated DNA Damage

The DNA molecule consists of two strands that twist around each other and form a double helical structure that carries the genetic instruction in all organisms. DNA integrity in a cell is of prime importance, and any threat to its stability will lead to mutations and, ultimately, cell death [75]. DNA damage and genomic instability are crucial factors in cancer progression and advancement [35]. When damage occurs, the DNA damage response (DDR) pathway takes control and decides whether to repair the damage or take the compromised cell toward programmed cell death through various DNA damage checkpoints [76]. AITC repressed cell cycle advancement in NSCLC cells via the replication-mediated DNA damage response (DDR), and the result was evident using immunofluorescence microscopy. Both A549 and H1299 cells showed 3 to 4 times increased FANCD2 foci and γH2AX foci after 6 h of treatment with 20 μM AITC compared to only DMSO-treated cells [32]. Bo et al. studied the possible effects of AITC on DNA integrity using the Comset assay. Augmented Comet tail length and enhanced DNA damage were observed in both MDA-MB-231 and MCF-7 after treatment with AITC for 24 h. Condensed and grainy chromatin, along with divided nuclei, were observed in both cell lines treated with AITC in a concentration-dependent manner compared to control cells [27]. Dose-dependent induction of AITC triggered DNA damage, as evidenced by the increased pChk2, H2AX, pChk1 (s317), cyclin B, and FANCD2 levels, including the breakage of double strands [43]. Comet and micronucleus assays revealed that DNA damage was observed in T24 cells with wild-type TP53 at all concentrations of AITC (0.005, 0.0625, 0.0725, 0.0825, 0.0925, 0.125, and 0.25 μM); however, in RT4 cells with the mutated TP53 gene, DNA damage was observed at only the three highest concentrations of AITC (0.0725, 0.0825, and 0.0925 μM) [23].

AITC was also assessed for its acute, sub-chronic, and short-term toxicity as well as its teratogenic effect in mice, hamsters, rabbits, and rats. The short-term toxicity studies indicated a thickness in the mucosal surface of the stomach and urinary bladder wall (observed only in male rats and mice); the adhesion of the stomach to the peritoneum was also observed. After receiving AITC, the rats and mice showed no observed adverse effect levels (NOAELs) in the range of 10 to 25 mg/kg (bw/day) in sub-chronic and short-term toxicity studies. However, AITC showed no significant signs of developmental toxicity when given orally to pregnant rabbits, rats, and hamsters at doses of about 12.3, 18.5, and 23.8 mg/kg (bw/day), respectively. AITC can be fetotoxic to mice when given in a dose higher than 6.0 mg/kg (bw/day) without revealing any teratogenic effects in them [77].

## 4. Delivery of AITC

AITC is an organosulfur compound that is a colorless to pale yellow liquid; it is miscible in most organic solvents and is partially soluble in water. We have earlier discussed the potential of AITC as an anticancer agent; however, the delivery of AITC has considerable challenges because of its poor solubility, instability, degradation, and offset toxicity. The oral dose of AITC is absorbed and metabolized through the mercapturic acid pathway, which is then excreted through urine. The data from in vivo studies showed that the concentration of AITC in urine and the tissues of the bladder was significantly higher than the amounts detected in plasma and other tissues. These findings indicate that AITC could be a reasonable anticancer agent for the treatment of bladder cancer [78]. Apart from that, AITC has shown anticancer activity against many other cancer types (discussed in the previous section); however, bioavailability is still a challenge. In this section, we summarize the previous work carried out for the delivery of AITC, which may increase the bioavailability of AITC for many other cancer types. A few encapsulation methods using nanotechnology have been reported in the literature to enhance the stability of AITC and targeted delivery.

### 4.1. AITC-Encapsulated Nanoemulsions

Li et al. reported oil-in-water (O/W) nanoemulsion-entrapped AITC to improve its aqueous solubility by using the emulsion-inversion-point method. Both Span 80 and Tween 80 were used as co-surfactants to disperse mineral oil droplets encapsulating AITC in the water phase. The hydrophilic–lipophilic balance was optimized to fabricate stable O/W nanodroplets, with size distributions ranging from 137 to 215 nm in diameter. It was found that the size and count rate of AITC-loaded nanoemulsified droplets decreased slightly (4–13%, depending on the co-surfactants-to-oil ratio) after storage for 6.5 months, ensuring enhanced stability. Their study further suggested that this method protects against the degradation of AITC, as they found 78% of AIT remained after 2 months of storage at 30 °C, thus showing improved chemical stability [79].

Chang et al. reported that AITC encapsulated with O/W emulsified nanoparticles (AITC-NPs) was prepared using the coacervation technique with the following composition—AITC: 18–22%, co-surfactants Tween-80/Tween-20/Span-80: 33/2/1, and water: 28–32%. The complex coacervation involved the formation of complex coacervation on the microemulsions’ surface through the electrostatic energy of a negatively charged sulfate group of AITC and positively charged alkali-treated gelatin. The synthesized AITC-NPs were considerably small, with sizes of just ~9.5 nm, and nearly spherical (evident by TEM imaging), and they showed enhanced anticancer and antioxidant properties. These AITC-NPs were quite stable, sustaining three freezing–thawing cycles and heat up to 110 °C. Moreover, compared to free AITC and empty NPs, AITC-NPs exhibited enhanced anticancer and anti-inflammatory responses against HT1376 bladder cancer cells and RAW 264.7 macrophage cells [25].

### 4.2. AIT-Loaded Polymeric Nanoparticles

Encinas-Basurto et al. studied the delivery of AITC by encapsulation into poly (lactic-co-glycolic acid) (PLGA) nanoparticles. The encapsulation of AITC with polymeric nanoparticles protects them from degradation, increases their half-life in blood, and helps to achieve a controlled and sustained release. They achieved AITC-loaded PLGA nanoparticles of ~200 nm in size by an emulsion solvent evaporation method using polyvinyl alcohol as a stabilizer. The in vitro studies showed a sustained release of AITC from AITC-PLGA NPs in MDA-MB-231 and HeLa cancer cell models, resulting in enhanced anticancer activity compared to free AITC [80]. The same group further studied the antibody-guided delivery of AITC-PLGA NPs by modifying the surface to covalently attach anti-EGFRs (epidermal growth factor receptors). EGFR is overexpressed on epithelial squamous carcinoma A431 cells and can be used as a checkpoint for targeted delivery to cancer cells. The AITC-PLGA NPs tagged with anti-EGFRs were tested in a co-culture experiment of A431 cells (EGFR overexpressed) and HeLa cells (EGFR less expressed) to test their targeting ability. The more specific localization of AITC-PLGA was found in A431 cells compared to a smaller number randomly distributed on HeLa cells. The study concluded that the enhanced anticancer activity of AITC-PLGA was due to the targeting of specific cancer cell receptors [81].

### 4.3. AITC-Conjugated Silicon Quantum Dots

Quantum dots (QDs) are semiconductor nanoparticles that are mostly used for bioimaging and therapeutic purposes. Silicon quantum dots (SiQDs) are a type of QD that is preferred for biomedicine because of their low toxicity. Liu et al. synthesized AITC-conjugated SiQDs (AITC-SiQDs) by first preparing hydrogen-terminated SiQDs and then reacting them with allyl bromide. When bromine was functionalized on SiQDs, it reacted with potassium thiocyanate to produce thiocyanate-functionalized 4 nm SiQDs with a spherical shape. The AITC-SiQDs showed the same anticancer activity as free AITC against liver cancer. However, AITC-SiQDs showed no biphasic effect (avoiding the low-dose stimulation effect) as shown by AITC in some cancer models. Moreover, the AITC-SiQDs can be used as imaging agents because of the intrinsic fluorescent properties of SiQDs. After 1-hour incubation of AITC-SiQDs in HepG2 cells, fluorescence was detected, and the peak signal was achieved after 12 h, showing a large number of accumulated AITC-SiQDs. The study concluded that the use of AITC-SiQDs instead of free AITC could minimize the biphasic effect and optimize the therapeutical potential of AITC [16].

### 4.4. In Situ Delivery of AITC Using the Sinigrin–Myrosinase System

Very recently, Tarar et al. reported the in-situ delivery of AITC using the sinigrin–myrosinase system to eradicate A549 lung cancer cells [82]. The gene sequence of myrosinase (MYR) and core streptavidin (coreSA) was cloned in pET30a(+) plasmids and produced MYR-coreSA fusion proteins using a T7 bacterial expression system. The A549 cells were biotinylated and decorated with myrosinase using streptavidin–biotin affinity and then treated with sinigrin to induce apoptosis. Considering the potential of cytotoxic AITC leaking from the aforementioned nanoparticles, the incorporation of a sinigrin–myrosinase system with nanocarriers for the delivery of AITC to tumor sites can be safer and more effective as sinigrin is completely safe and biologically inactive if it is leaked during delivery.

## 5. Conclusions

Cancer is one of the leading causes of death globally, with millions of deaths annually, and this trend is projected to increase further in the next decade. Chemotherapy is widely used to treat cancer. Since cancer cells are deficient in several regulatory functions and divide abnormally, this makes them susceptible to chemotherapy drugs. Several chemotherapy drugs have been developed, but the toxicity of these drugs has unwanted side effects, i.e., nausea, hair loss, decreased red blood cell count, etc. Despite considerable efforts, cancer still is an aggressive killer. Therefore, there is a need to develop alternative, effective, and affordable anticancer therapeutics. Medicinal plants have been used for centuries for therapeutic purposes, including anticancer treatment. Cruciferous vegetables are one such example, and consumption of them is linked to a decrease in the risk of cancer. Studies suggest that the anticancer activity is attributed mainly to ITCs (isothiocyanates) present in these plants. AITC is a type of ITC that is abundantly present in broccoli, mustard seed, wasabi, cabbage, cauliflower, etc. The anticancer activity of AITC has been well established and reviewed in this article, including the possible anticancer mechanisms. However, the clinical application of AITC is hindered by its volatility, instability, low solubility, and low viability, which makes the delivery of AITC a challenging task. Some studies have addressed this issue by delivering AITC encapsulated in nanoparticles, but further research is required for translational medicine. In one of our previous studies, we used sinigrin as a prodrug to produce AITC in situ, which provides a potential approach for anticancer treatment.

## Figures and Tables

**Table 1:** Summary of the anticancer activity of AITC.

Cancer Cell Type	In Vivo/In Vitro Model, Cell Type	Concentration Range	Treatment Time	IC_50_/GC_50_/EC_50_ Values	References
Non-metastatic and metastatic melanoma cells	In vitro model, A375, B16F-10, VMM1, Hs294T, A431, HaCaT cells	10 μM	24 and 48 h	-	Mitsiogianni et al., 2021 [9]
Human prostate cancer cells	In vitro, PC-3 cells	50–100 μM	24 h and 48 h	Dose-dependent	Xu et al., 2005 [10]
	In vitro, PC-3 cells, In vivo, PC-3 xenografts model	PC-3 xenografts (Bolus i.p. injection of 10 mmol AITC), PC-3 cells (0–9 μM)	PC-3 xenografts (three times per week), PC-3 cells (10 days)	IC_50_ of ~2.2 μM for PC-3 cells	Srivastava et al., 2003 [11,12]
	In vitro, PC-3 (androgen-independent) and LNCaP (androgen-dependent), PrEC cells	20 μM AITC	24, 48, or 72 h	IC_50_ of ~15–17 μM	Xiao et al., 2003 [11]
	In vitro, PC3 (CRL-1435), CWR22Rv1 (Rv1; CRL-2505), PrECs	0–80 μM AITC	24 h to 3 days	-	Chen et al., 2018 [13]
Human cisplatin-resistant oral cancer cells	In vitro, CAL27 (CAR cells)	0, 10, 20, 30, and 40 μM	24 and 48 h	Dose-dependent	Chang et al., 2020 [13]
Human leukemia cell	In vitro, HL60 (p53-), ML1(p53+)	100 nM–50 μM	48 h	GC50 for ML-1 cells (2.41–3.22 μM), GC50 for HL60 cells (1.49–3.22 μM)	Xu et al., 2000 [14]
Human and mouse hepatoma cells	In vitro, mouse Hepa1c1c7 cells	AITC (0.1–20 μM), AITC-NAC (1 and 20 μM)	24 h	Dose-dependent	Hwang et al., 2005 [15]
	In vitro, HepG2, HHL5, murine MII perivascular M2 cells	AITC (0–320 μM), AITC-SiQDs (0–40 μM)	0 to 24 h	Dose-dependent	Liu et al., 2018 [16]
	In vitro, SK-Hep l cells	AITC (0–20 μM), NAC-AITC (0–20 μM)	24 and 72 h	Dose-dependent	Hwang et al., 2006 [17]
Human brain malignant glioma cells	In vitro, GBM 8401 cells	0.5, 1, 5, 10, and 20 μM	24 h	IC_50_ (9.25 ± 0.69 μM)	Chen et al., 2010 [18]
Human bladder cancer cells	In vitro, UM-UC-3 cells, AY-27 cells, In vivo, orthotopic AY27 cells in a female F344 rat model	13 and 26 μM for in vitro model, 9 or 90 μmol/kg bw* (71.5 or 715 mg MSP-1 per kg bw*) for in vivo model	In vitro model (24 and 72 h); in vivo (once daily for 3 weeks, started 1 day after cancer cell inoculation)	IC_50_ values of 10.8 and 8.6 μM for UM-UC-3, and AY-27 cells, respectively, 85.8 and 68.3 µg MSP- 1 per ml culture medium, respectively	Bhattacharya et al., 2010 [19]
	In vitro, UM-UC-3 cells, UM-UC-6 cells, and T24 cells	0, 7.5, 15, and 30 μM	24 h	Dose-dependent	Geng et al., 2011 [20]
	In vitro, UM-UC-3 cells, AY-27, HUCs, in vivo, AY-27 cells were simultaneously inoculated both orthotopically and subcutaneously in a rat	In vitro (1–100 μM), In vivo (0, 10, 25, 50, 300 μmol/kg)	In vivo (once daily), in vitro (72 h)	IC_50_ of 2.7, 3.3, and 69.4 μM for UM-UC-3, AY-27, and HUC cells, respectively	Bhattacharya et al., 2009 [21]
	In vitro, UM-UC-3, AY-27 cell line; in vivo, female F344 rats	In vitro (NAC-AITC at 15 μM in UM-UC-3 and AY-27 cells); in vivo (at 10 μmol/kg body wt orally in rat bladder cancer model)	In vitro (24 h), in vivo (initiated 1 day after AY-27 cell inoculation and continued for 3 weeks)	IC_50_ of 7.4 and 9.1 μM for UM-UC-3, AY-27 cells, respectively	Bhattacharya et al., 2011 [22]
	In vitro, RT4 cell lines with a wild-type TP53 gene, T24 cell line with the TP53 allele	5.0, 62.5, 72.5, 82.5, and 92.5 μM	3 h	IC_50_ values of 310 and 350 μM for RT4 and T24 cells, respectively	Sávio et al., 2014 [23,24]
	In vitro HT1376 cells	AITC-equivalent doses of AITC-NPs (0.25, 0.50, 1.00, 1.43, 2.00, 2.50, and 3.34 g/L)	4 to 24 h	IC_50_ of 1.15 g/L of AITC-NPs or 35.87 mg/L of AITC	Chang et al., 2018 [25]
Macrophages	In vitro, RAW 264.7 cells	AITC-equivalent doses of AITC-NPs (0.25, 0.50, 1.00, 1.43, 2.00, 2.50, and 3.34 g/L)	4 to 24 h	IC_50_ of 0.89 g/L of AITC-NPs and 31.1 mg/L of AITC	Chang et al., 2018 [25]
Human breast adenocarcinoma cells	In vitro MDA-MB-468 cells	0,5, 10, and 20 μM	24 and 48 h	IC_50_ of 10.26 ± 1.31 μM	Tsai et al., 2012 [26]
Human breast cancer Cells	In vitro MCF-7 (estrogen receptor positive), MDA-MB-231 (estrogen receptor negative) cells	0, 1.5625, 3.125, 6.25, 12.5, and 25 μM	48 h	Dose-dependent	Bo et al., 2016 [27]
Human and mouse mammary carcinoma	In vivo, female Sprague–Dawley rats	10, 20, and 40 mg/kg bw*	once a day by starting one week before the exposure to the carcinogen	Dose-dependent	Thangarasu et al., 2018 and 2015 [28,29,30]
	In vitro, EAT (Ehrlich ascites tumor) cells; in vivo, EAT cells were injected into 8-week-old Swiss Albino mice	1, 5, 10, and 15 μM	24, 48, and 72 h	Dose-dependent	Kumar et al., 2009 [31]
Human non-small cell lung cancer (NSCLC) cells	In vitro, A549 cells, H1299, HBECs cells	5, 10, and 20 μM	6, 16, 24, and 48 h	IC_50_ values of 10 and 5 μM for A549 and H1299 cells, respectively	Tripathi et al., 2015 [32]
	In vitro, A549 cells	AITC (2.5–12.5 μM), AITC + SFN (6.25 μM AITC with 5 μM SFN)	72 h	IC_50_ values of 12.64 μM for AITC, IC_50_ of 5.53 μM AITC, and 4.43 μM SFN for the combined treatment	Rakariyatham et al., 2019 [33]
Human cervical cancer cells	In vitro, HeLa cells	0, 5, 15, and 45 μM AITC	24, 48, and 72 h	Dose-dependent	Qin et al., 2017 [34]
Human colorectal adenocarcinoma cells	In vitro, HT-29 cells	12 μM	7 and 24 h	-	Smith et al., 2004 [35]
	In vitro, HT29 cells	5 and 10 μM of AITC	24 h	Dose-dependent	Lai et al., 2013 [36]
	In vitro, HT29 cells	1.2–1.6 µg/ml	24 h	Dose-dependent	Musk et al., 1993 [37]
	In vitro, Hs68, Caco-2, COLO 201, SW620 cells; in vivo, SW620 xenograft	In vitro (0–150 μM), SW620 xenografts (5 and 10 μmol)	24 and 72 h	Dose-dependent	Lau et al., 2010 [38]
Malignant melanoma cells	In vitro, A375, B16-F10, VMM1, Hs294T, A431, HaCaT cells	2.5 and 50 μM	24 and 48 h	EC_50_ of 15.6 and 21.7 μM for A375 and Hs294T cells, respectively, after 24 h and 12.0, 43.4, 21.3, and 14.9 μM for A375, A431, Hs294T, and B16-F10 cells, respectively, after 48 h	Mitsiogianni et al., 2020 [39]
Renal carcinoma cell line (RCC)	In vitro, GRC-1 cells	0, 7.5, 15, and 30 μM	24, 48, and 72 h	Dose-dependent	Jiang et al., 2016 [40]

Footnote: bw* donates body weight.
